# 4-Carb­oxy­pyridin-1-ium 2,4,5-tri­carb­oxy­benzoate monohydrate

**DOI:** 10.1107/S1600536813016437

**Published:** 2013-06-19

**Authors:** Hadi D. Arman, Edward R. T. Tiekink

**Affiliations:** a Department of Chemistry, The University of Texas at San Antonio, One UTSA Circle, San Antonio, Texas 78249-0698, USA; b Department of Chemistry, University of Malaya, 50603 Kuala Lumpur, Malaysia

## Abstract

The title hydrated salt, C_6_H_6_NO_2_^+^·C_10_H_5_O_8_^−^·H_2_O, was isolated from the 1:1 cocrystallization of benzene-1,2,4,5-tetra­carb­oxy­lic acid and isonicotinic acid in ethanol solution. In the crystal, the cation is close to planar [r.m.s. deviation = 0.085 Å for the nine fitted atoms; the C—C—C—O(carbon­yl) torsion angle = −8.7 (4)°], but twists are evident in the anion, with all but the carb­oxy­lic acid group diagonally opposite the carboxyl­ate group being significantly twisted out of the plane of the benzene ring [C—C—C—O(carbon­yl) torsion angles = −118.1 (2), −157.6 (2), 4.3 (3) and 77.3 (3)°]. In the crystal, the ions and water mol­ecules are consolidated into a three-dimensional architecture by O—H⋯O and N—H⋯O hydrogen bonding along with C—H⋯O inter­actions.

## Related literature

For background to pharmaceutical co-crystals, see: Almarsson & Zaworotko (2004 ▶). For related co-crystallization studies on 1,2,4,5-benzene­tetra­carb­oxy­lic acid, see: Arman & Tiekink (2013*a* ▶,*b* ▶). For the structure of isonicotinic acid, see: Takusagawa & Shimada (1976 ▶). For the structure of the analogous salt formed from nicotinic acid, see: Dos Santos *et al.* (2012 ▶). For the calculation of p*K*_a_ values, see: Chemaxon (2009 ▶).
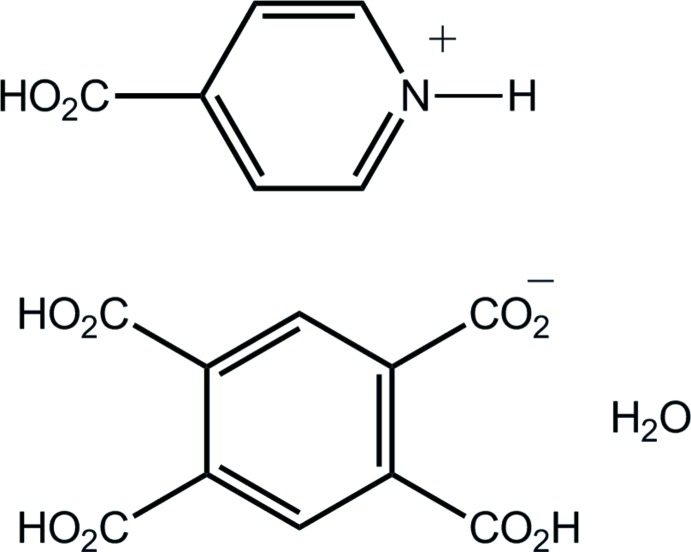


## Experimental

### 

#### Crystal data

C_6_H_6_NO_2_^+^·C_10_H_5_O_8_^−^·H_2_O
*M* *_r_* = 395.27
Triclinic, 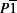

*a* = 9.724 (2) Å
*b* = 10.007 (2) Å
*c* = 10.755 (2) Å
α = 99.56 (1)°
β = 114.667 (8)°
γ = 110.283 (9)°
*V* = 830.7 (3) Å^3^
*Z* = 2
Mo *K*α radiation
μ = 0.14 mm^−1^
*T* = 98 K
0.33 × 0.25 × 0.20 mm

#### Data collection

Rigaku AFC12/SATURN724 diffractometer
Absorption correction: multi-scan (*ABSCOR*; Higashi, 1995 ▶) *T*_min_ = 0.807, *T*_max_ = 1.000
5233 measured reflections
3414 independent reflections
3200 reflections with *I* > 2σ(*I*)
*R*_int_ = 0.020

#### Refinement

*R*[*F* ^2^ > 2σ(*F* ^2^)] = 0.056
*wR*(*F* ^2^) = 0.133
*S* = 1.17
3414 reflections
281 parameters
8 restraints
H atoms treated by a mixture of independent and constrained refinement
Δρ_max_ = 0.34 e Å^−3^
Δρ_min_ = −0.27 e Å^−3^

### 

Data collection: *CrystalClear* (Molecular Structure Corporation & Rigaku, 2005 ▶); cell refinement: *CrystalClear*; data reduction: *CrystalClear*; program(s) used to solve structure: *SHELXS97* (Sheldrick, 2008 ▶); program(s) used to refine structure: *SHELXL97* (Sheldrick, 2008 ▶); molecular graphics: *ORTEP-3 for Windows* (Farrugia, 2012 ▶) and *DIAMOND* (Brandenburg, 2006 ▶); software used to prepare material for publication: *publCIF* (Westrip, 2010 ▶).

## Supplementary Material

Crystal structure: contains datablock(s) global, I. DOI: 10.1107/S1600536813016437/hb7093sup1.cif

Structure factors: contains datablock(s) I. DOI: 10.1107/S1600536813016437/hb7093Isup2.hkl

Supplementary material file. DOI: 10.1107/S1600536813016437/hb7093Isup3.cml

Additional supplementary materials:  crystallographic information; 3D view; checkCIF report

## Figures and Tables

**Table 1:** Hydrogen-bond geometry (Å, °)

*D*—H⋯*A*	*D*—H	H⋯*A*	*D*⋯*A*	*D*—H⋯*A*
O4—H4*O*⋯O3^i^	0.84 (3)	1.82 (3)	2.654 (3)	176 (3)
O6—H6*O*⋯O1^ii^	0.85 (5)	1.69 (5)	2.534 (3)	174 (5)
O8—H8*O*⋯O1*W*	0.85 (5)	1.79 (5)	2.634 (3)	171 (6)
O10—H10*O*⋯O2^iii^	0.85 (4)	1.78 (4)	2.625 (3)	172 (4)
N1—H1*N*⋯O5^iv^	0.89 (4)	1.86 (4)	2.711 (3)	160 (4)
O1*W*—H1*W*⋯O2^v^	0.85 (2)	2.16 (2)	2.957 (3)	156 (3)
O1*W*—H2*W*⋯O2^iii^	0.85 (1)	2.05 (2)	2.853 (3)	158 (4)
C6—H6⋯O7^v^	0.95	2.40	3.267 (3)	151
C12—H12⋯O9^vi^	0.95	2.35	3.179 (4)	146
C14—H14⋯O6^vii^	0.95	2.40	3.300 (4)	159

Symmetry codes: (i) 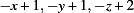; (ii) 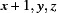; (iii) 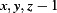; (iv) 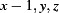; (v) 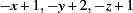; (vi) 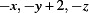; (vii) 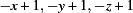.

**Fig. 1.**
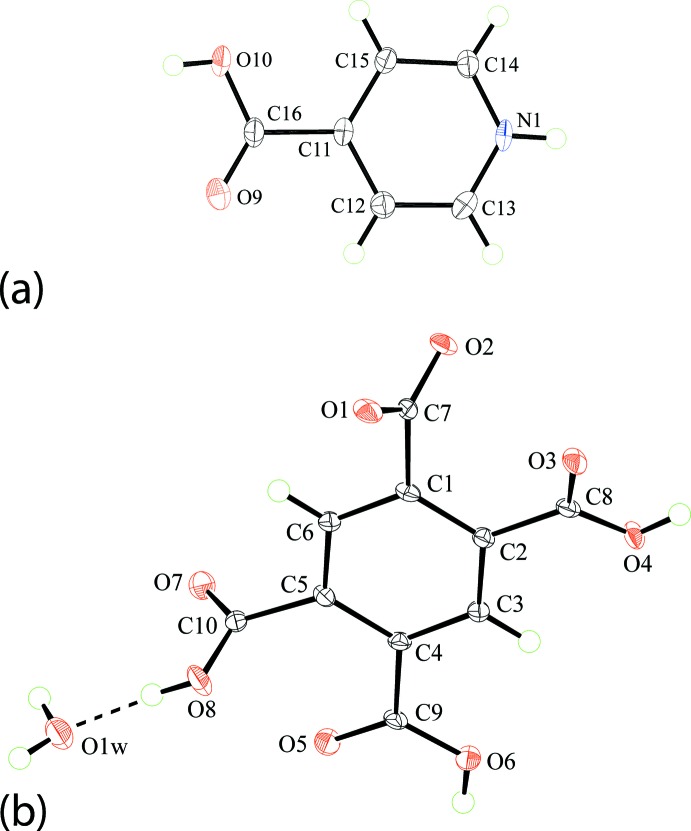
Molecular structures of (a) the 4-carboxypyridin-1-ium cation and (b) the hydroxy(2,4,5-tricarboxyphenyl)methanolate anion and water molecule (hydrogen bond shown as a dashed line) in (I), showing displacement ellipsoids at the 50% probability level.

**Fig. 2.**
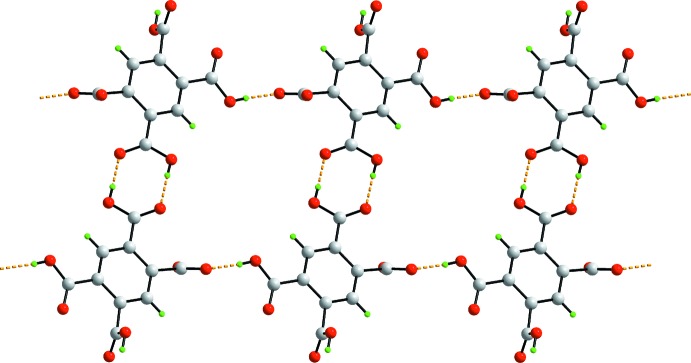
Supramolecular tape mediated by O—H···O hydrogen bonding between anions. The O—H···O hydrogen bonds are shown as orange dashed lines.

**Fig. 3.**
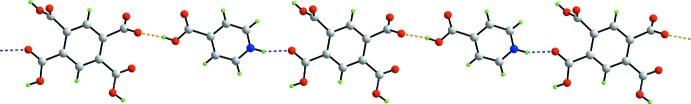
Supramolecular chain mediated by N—H···O hydrogen bonding between cations and anions. The N—H···O hydrogen bonds are shown as blue dashed lines.

**Fig. 4.**
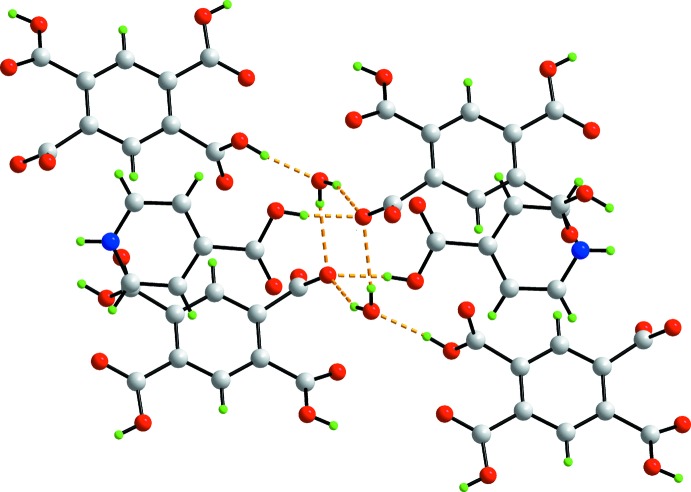
Detail of the hydrogen bonding involving the trifurcated carboxylate-O2 atom and water molecule of solvation.

**Fig. 5.**
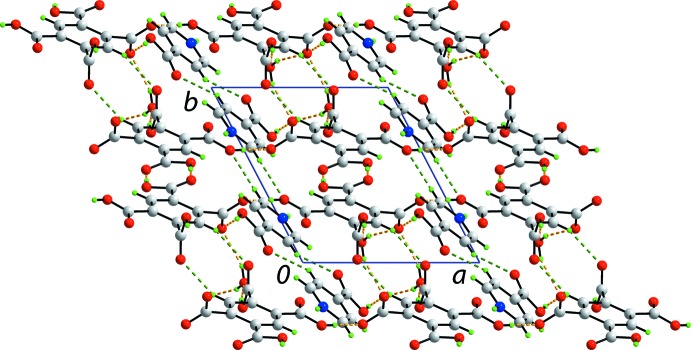
Unit-cell contents in (I) viewed in projection down the c axis. The C—H···O interactions are shown as green dashed lines.

## supplementary crystallographic information

### Comment

Interest in co-crystallization experiments stems largely from potential applications in the pharmaceutical industry (Almarsson & Zaworotko, 2004). The title salt hydrate, (I), was isolated during an investigation of co-crystallization experiments with 1,2,4,5-benzenetetracarboxylic acid (pyromellitic acid; LH_4_) and various pyridyl derivatives (Arman & Tiekink, 2013*a*; Arman & Tiekink, 2013*b*).

The 1:1 co-crystallization of pyromellitic acid and isonicotinic acid in an ethanol solution afforded a salt hydrate, (I), there being one complete molecule of the 4-carboxypyridin-1-ium cation, the hydroxy(2,4,5-tricarboxyphenyl)methanolate anion and a water molecule in the asymmetric unit. Confirmation of protonation of isonicotinic acid during crystallization is found in the nature of hydrogen bonding involving the pyridinium cation (see below), and the widening of the C13—N1—C14 angle to 123.1 (2)° *cf*. 118.9 (2)° in the structure of isonicotinic acid itself (Takusagawa & Shimada, 1976). Further, the disparity in the C—O bond lengths of the carboxylic acid residue, Δ(C—O) = [(C—O)_long_ - (C—O)_short_] = 0.10 Å, confirms that isonicotinic acid has been protonated at N1, rather than existing in a zwitterionic form, with equivalent C—O bonds. Complementing this evidence, in the anion, the Δ(C—O) value for the O1-carboxylate group is 0.01 Å, indicating considerable delocalization of electron density over the CO_2_ atoms, compared to values in the range 0.07 to 0.11 Å for the remaining carboxylic acids.

In the cation, the carboxylic acid residue is approximately co-planar with the pyridyl ring to which it is attached as seen in the C12—C11—C16—O9 torsion angle of -8.7 (4)°. Indeed, the entire cation is approximately planar with the r.m.s. deviation = 0.085 Å for the nine fitted atoms. By contrast, anion is non-planar with the carboxylic acid diagonally opposite the carboxylate group being co-planar to the phenyl ring [*C*—*C*—*C*—*O*(carbonyl) torsion angle = 4.3 (3)°] but the remaining carboxylic acids and carboxylate residues deviate significantly [*C*—*C*—*C*—*O*(carbonyl) torsion angles =-118.1 (2), -157.6 (2) and 77.3 (3)°].

The three-dimensional crystal structure of (I) is consolidated by O—H···O and N—H···O hydrogen bonding involving all three components of the structure; geometric parameters characterizing these are summarized in Table 1. Two distinct supramolecular aggregation patterns are clearly discerned, with the first of these being a tape along [1 0 0] comprising hydroxy(2,4,5-tricarboxyphenyl)methanolate anions, Fig. 2, which is sustained by centrosymmetric eight-membered {···O=COH}_2_ synthons involving the O3-carboxylic acid and orthogonal O6—H···O1 hydrogen bonds. A supramolecular chain containing alternating cations and anions is also found, Fig. 3. This is aligned along [1 0 - 1] and is stabilized by O10—H···O2 and N1—H···O5 hydrogen bonds. The aforementioned are connected into a supramolecular layer in the *ac* plane. As seen in Fig. 4, the centrosymmetrically related water molecules are bridged by carboxylate-O2 atoms to form an eight-membered {···HOH···O}_2_ ring. The water-O1w atom also accepts a hydrogen bond from the O8—H hydroxyl group. Also evident from the image in Fig. 4 is the critical role played by the trifurcated carboxylate-O2 atom in stabilizing the crystal structure. Several of the oxygen atoms not involved in formal hydrogen bonding interactions participate in C—H···O interactions to consolidate the crystal packing, Fig. 5.

While salt (I) was characterized from the 1:1 co-crystallization of LH_4_ with isonicoinic acid in an ethanol solution, the analogous experiment with nicotinic [NA] acid gave a salt of composition [NAH]_2_[LH_2_] (Dos Santos, *et al.*, 2012). While protonation of NA is not surprising the fact that *L*H_4_ has been doubly deprotonated correlates nicely with the increased basicity of the nitrogen atom in NA *cf*. isonicotinic acid, *i.e*. calculated p*K*_a_ = 4.19 and 2.35, respectively (Chemaxon, 2009).

### Experimental

Crystals of the title salt hydrate were harvested from an ethanol (10 ml) solution containing a 1:1 molar ratio of pyromellitic acid (Sigma-Aldrich; 6 mg) and isonicoinic acid (Sigma-Aldrich; 3 mg).

### Refinement

C-bound H-atoms were placed in calculated positions (C—H 0.95 Å) and were included in the refinement in the riding model approximation with *U*_iso_(H) set to 1.2*U*_eq_(C). The O—H and N—H atoms were located in difference maps and were refined with O—H = 0.84±0.01 Å and N—H = 0.88±0.01 Å, respectively, and with *U*_iso_(H) = 1.5*U*_eq_(O) and *U*_iso_(H) = 1.2*U*_eq_(N), respectively.

### Crystal data

**Table:**

C_6_H_6_NO_2_^+^·C_10_H_5_O_8_^−^·H_2_O	*Z* = 2
*M**_r_* = 395.27	*F*(000) = 408
Triclinic, *P*1	*D*_x_ = 1.580 Mg m^−^^3^
Hall symbol: -P 1	Mo *K*α radiation, λ = 0.71073 Å
*a* = 9.724 (2) Å	Cell parameters from 3944 reflections
*b* = 10.007 (2) Å	θ = 2.3–29.8°
*c* = 10.755 (2) Å	µ = 0.14 mm^−^^1^
α = 99.56 (1)°	*T* = 98 K
β = 114.667 (8)°	Block, colourless
γ = 110.283 (9)°	0.33 × 0.25 × 0.20 mm
*V* = 830.7 (3) Å^3^	

### Data collection

**Table:**

Rigaku AFC12K/SATURN724 diffractometer	3414 independent reflections
Radiation source: fine-focus sealed tube	3200 reflections with *I* > 2σ(*I*)
Graphite monochromator	*R*_int_ = 0.020
ω scans	θ_max_ = 26.5°, θ_min_ = 2.3°
Absorption correction: multi-scan (*ABSCOR*; Higashi, 1995)	*h* = −11→12
*T*_min_ = 0.807, *T*_max_ = 1.000	*k* = −12→12
5233 measured reflections	*l* = −13→9

### Refinement

**Table:**

Refinement on *F*^2^	Primary atom site location: structure-invariant direct methods
Least-squares matrix: full	Secondary atom site location: difference Fourier map
*R*[*F*^2^ > 2σ(*F*^2^)] = 0.056	Hydrogen site location: inferred from neighbouring sites
*wR*(*F*^2^) = 0.133	H atoms treated by a mixture of independent and constrained refinement
*S* = 1.17	*w* = 1/[σ^2^(*F*_o_^2^) + (0.0449*P*)^2^ + 1.2826*P*] where *P* = (*F*_o_^2^ + 2*F*_c_^2^)/3
3414 reflections	(Δ/σ)_max_ < 0.001
281 parameters	Δρ_max_ = 0.34 e Å^−^^3^
8 restraints	Δρ_min_ = −0.27 e Å^−^^3^

### Special details

**Table:**

Geometry. All s.u.'s (except the s.u. in the dihedral angle between two l.s. planes) are estimated using the full covariance matrix. The cell s.u.'s are taken into account individually in the estimation of s.u.'s in distances, angles and torsion angles; correlations between s.u.'s in cell parameters are only used when they are defined by crystal symmetry. An approximate (isotropic) treatment of cell s.u.'s is used for estimating s.u.'s involving l.s. planes.
Refinement. Refinement of *F*^2^ against ALL reflections. The weighted *R*-factor *wR* and goodness of fit *S* are based on *F*^2^, conventional *R*-factors *R* are based on *F*, with *F* set to zero for negative *F*^2^. The threshold expression of *F*^2^ > 2σ(*F*^2^) is used only for calculating *R*-factors(gt) *etc*. and is not relevant to the choice of reflections for refinement. *R*-factors based on *F*^2^ are statistically about twice as large as those based on *F*, and *R*- factors based on ALL data will be even larger.

### Fractional atomic coordinates and isotropic or equivalent isotropic displacement parameters (Å^2^)

**Table:**

	*x*	*y*	*z*	*U*_iso_*/*U*_eq_	
O1	0.1555 (2)	0.6476 (2)	0.60109 (18)	0.0198 (4)	
O2	0.3582 (2)	0.80549 (19)	0.83497 (17)	0.0192 (4)	
O3	0.3881 (2)	0.53016 (19)	0.85274 (17)	0.0180 (4)	
O4	0.6609 (2)	0.56930 (19)	0.94793 (17)	0.0164 (3)	
H4O	0.642 (4)	0.534 (4)	1.008 (3)	0.037 (9)*	
O5	0.8655 (2)	0.7742 (2)	0.5022 (2)	0.0235 (4)	
O6	0.9197 (2)	0.6410 (2)	0.64907 (19)	0.0210 (4)	
H6O	0.994 (4)	0.643 (5)	0.627 (5)	0.076 (15)*	
O7	0.6828 (2)	0.98272 (19)	0.46806 (18)	0.0198 (4)	
O8	0.5263 (2)	0.7429 (2)	0.29663 (18)	0.0219 (4)	
H8O	0.542 (6)	0.788 (5)	0.240 (4)	0.077 (15)*	
O9	0.1620 (3)	0.9309 (3)	−0.0204 (3)	0.0409 (6)	
O10	0.1966 (2)	0.7184 (2)	−0.0264 (2)	0.0251 (4)	
H10O	0.243 (4)	0.751 (4)	−0.074 (3)	0.039 (9)*	
O1W	0.6084 (2)	0.8838 (2)	0.1330 (2)	0.0251 (4)	
H1W	0.645 (4)	0.9798 (13)	0.166 (3)	0.042 (10)*	
H2W	0.549 (4)	0.849 (3)	0.0401 (11)	0.058 (12)*	
N1	−0.0175 (3)	0.7469 (2)	0.3153 (2)	0.0187 (4)	
H1N	−0.054 (4)	0.736 (4)	0.378 (3)	0.037 (9)*	
C1	0.4428 (3)	0.7087 (2)	0.6751 (2)	0.0129 (4)	
C2	0.5480 (3)	0.6444 (2)	0.7461 (2)	0.0126 (4)	
C3	0.6741 (3)	0.6448 (2)	0.7146 (2)	0.0127 (4)	
H3	0.7450	0.6024	0.7630	0.015*	
C4	0.6972 (3)	0.7066 (2)	0.6129 (2)	0.0123 (4)	
C5	0.5920 (3)	0.7703 (2)	0.5415 (2)	0.0128 (4)	
C6	0.4668 (3)	0.7715 (2)	0.5742 (2)	0.0136 (4)	
H6	0.3973	0.8155	0.5272	0.016*	
C7	0.3075 (3)	0.7199 (3)	0.7072 (2)	0.0138 (4)	
C8	0.5237 (3)	0.5753 (2)	0.8532 (2)	0.0134 (4)	
C9	0.8360 (3)	0.7090 (2)	0.5826 (2)	0.0131 (4)	
C10	0.6099 (3)	0.8443 (2)	0.4328 (2)	0.0132 (4)	
C11	0.0918 (3)	0.7909 (3)	0.1229 (2)	0.0171 (5)	
C12	0.0210 (3)	0.8792 (3)	0.1604 (3)	0.0202 (5)	
H12	0.0106	0.9548	0.1194	0.024*	
C13	−0.0339 (3)	0.8546 (3)	0.2584 (3)	0.0215 (5)	
H13	−0.0827	0.9131	0.2853	0.026*	
C14	0.0488 (3)	0.6591 (3)	0.2804 (3)	0.0199 (5)	
H14	0.0573	0.5842	0.3229	0.024*	
C15	0.1047 (3)	0.6786 (3)	0.1820 (3)	0.0194 (5)	
H15	0.1508	0.6169	0.1555	0.023*	
C16	0.1542 (3)	0.8213 (3)	0.0175 (3)	0.0222 (5)	

### Atomic displacement parameters (Å^2^)

**Table:**

	*U*^11^	*U*^22^	*U*^33^	*U*^12^	*U*^13^	*U*^23^
O1	0.0155 (8)	0.0324 (9)	0.0186 (8)	0.0140 (7)	0.0122 (7)	0.0092 (7)
O2	0.0233 (9)	0.0281 (9)	0.0162 (8)	0.0155 (7)	0.0150 (7)	0.0089 (7)
O3	0.0172 (8)	0.0267 (9)	0.0195 (8)	0.0122 (7)	0.0138 (7)	0.0133 (7)
O4	0.0156 (8)	0.0248 (8)	0.0150 (8)	0.0103 (7)	0.0104 (7)	0.0122 (7)
O5	0.0293 (10)	0.0366 (10)	0.0315 (10)	0.0243 (8)	0.0261 (8)	0.0242 (8)
O6	0.0232 (9)	0.0355 (10)	0.0279 (9)	0.0227 (8)	0.0213 (8)	0.0218 (8)
O7	0.0232 (9)	0.0193 (8)	0.0222 (9)	0.0103 (7)	0.0146 (7)	0.0108 (7)
O8	0.0294 (9)	0.0236 (9)	0.0135 (8)	0.0100 (8)	0.0128 (7)	0.0083 (7)
O9	0.0678 (16)	0.0473 (13)	0.0561 (14)	0.0395 (12)	0.0537 (13)	0.0393 (11)
O10	0.0327 (10)	0.0353 (10)	0.0244 (9)	0.0202 (9)	0.0230 (8)	0.0163 (8)
O1W	0.0305 (10)	0.0285 (10)	0.0195 (9)	0.0134 (8)	0.0150 (8)	0.0113 (8)
N1	0.0184 (10)	0.0241 (10)	0.0143 (9)	0.0065 (8)	0.0121 (8)	0.0060 (8)
C1	0.0124 (10)	0.0155 (10)	0.0121 (10)	0.0061 (8)	0.0083 (8)	0.0034 (8)
C2	0.0130 (10)	0.0158 (10)	0.0115 (10)	0.0078 (8)	0.0074 (8)	0.0050 (8)
C3	0.0129 (10)	0.0156 (10)	0.0133 (10)	0.0081 (8)	0.0081 (8)	0.0065 (8)
C4	0.0127 (10)	0.0146 (10)	0.0133 (10)	0.0076 (8)	0.0088 (8)	0.0046 (8)
C5	0.0136 (10)	0.0155 (10)	0.0102 (9)	0.0067 (8)	0.0070 (8)	0.0045 (8)
C6	0.0145 (10)	0.0183 (10)	0.0142 (10)	0.0106 (9)	0.0094 (9)	0.0073 (8)
C7	0.0178 (11)	0.0191 (11)	0.0156 (10)	0.0123 (9)	0.0129 (9)	0.0111 (9)
C8	0.0158 (10)	0.0150 (10)	0.0130 (10)	0.0079 (8)	0.0101 (9)	0.0044 (8)
C9	0.0127 (10)	0.0190 (11)	0.0133 (10)	0.0088 (9)	0.0098 (8)	0.0066 (8)
C10	0.0118 (10)	0.0198 (11)	0.0148 (10)	0.0107 (9)	0.0088 (8)	0.0089 (9)
C11	0.0172 (11)	0.0208 (11)	0.0147 (10)	0.0075 (9)	0.0104 (9)	0.0064 (9)
C12	0.0212 (12)	0.0207 (11)	0.0202 (11)	0.0093 (10)	0.0118 (10)	0.0081 (9)
C13	0.0213 (12)	0.0244 (12)	0.0220 (12)	0.0117 (10)	0.0138 (10)	0.0057 (10)
C14	0.0217 (12)	0.0233 (12)	0.0187 (11)	0.0103 (10)	0.0129 (10)	0.0097 (9)
C15	0.0218 (12)	0.0255 (12)	0.0187 (11)	0.0140 (10)	0.0135 (10)	0.0098 (10)
C16	0.0260 (13)	0.0313 (13)	0.0200 (11)	0.0160 (11)	0.0168 (10)	0.0135 (10)

### Geometric parameters (Å, º)

**Table:**

O1—C7	1.260 (3)	C1—C2	1.418 (3)
O2—C7	1.271 (3)	C1—C7	1.528 (3)
O3—C8	1.234 (3)	C2—C3	1.400 (3)
O4—C8	1.327 (3)	C2—C8	1.497 (3)
O4—H4O	0.845 (10)	C3—C4	1.401 (3)
O5—C9	1.232 (3)	C3—H3	0.9500
O6—C9	1.303 (3)	C4—C5	1.415 (3)
O6—H6O	0.847 (10)	C4—C9	1.509 (3)
O7—C10	1.219 (3)	C5—C6	1.404 (3)
O8—C10	1.333 (3)	C5—C10	1.521 (3)
O8—H8O	0.846 (10)	C6—H6	0.9500
O9—C16	1.221 (3)	C11—C15	1.397 (3)
O10—C16	1.323 (3)	C11—C12	1.398 (3)
O10—H10O	0.845 (10)	C11—C16	1.519 (3)
O1W—H1W	0.849 (10)	C12—C13	1.386 (3)
O1W—H2W	0.847 (10)	C12—H12	0.9500
N1—C13	1.347 (3)	C13—H13	0.9500
N1—C14	1.347 (3)	C14—C15	1.389 (3)
N1—H1N	0.885 (10)	C14—H14	0.9500
C1—C6	1.401 (3)	C15—H15	0.9500
			
C8—O4—H4O	110 (2)	O2—C7—C1	117.70 (19)
C9—O6—H6O	111 (3)	O3—C8—O4	124.0 (2)
C10—O8—H8O	110 (3)	O3—C8—C2	122.2 (2)
C16—O10—H10O	106 (2)	O4—C8—C2	113.81 (18)
H1W—O1W—H2W	109.7 (16)	O5—C9—O6	124.6 (2)
C13—N1—C14	123.1 (2)	O5—C9—C4	120.44 (19)
C13—N1—H1N	116 (2)	O6—C9—C4	114.93 (18)
C14—N1—H1N	121 (2)	O7—C10—O8	125.1 (2)
C6—C1—C2	119.26 (19)	O7—C10—C5	121.98 (19)
C6—C1—C7	117.27 (19)	O8—C10—C5	112.69 (18)
C2—C1—C7	123.42 (19)	C15—C11—C12	120.4 (2)
C3—C2—C1	119.65 (19)	C15—C11—C16	121.5 (2)
C3—C2—C8	120.08 (19)	C12—C11—C16	118.1 (2)
C1—C2—C8	120.27 (19)	C13—C12—C11	118.8 (2)
C2—C3—C4	121.0 (2)	C13—C12—H12	120.6
C2—C3—H3	119.5	C11—C12—H12	120.6
C4—C3—H3	119.5	N1—C13—C12	119.5 (2)
C3—C4—C5	119.42 (19)	N1—C13—H13	120.2
C3—C4—C9	120.37 (19)	C12—C13—H13	120.2
C5—C4—C9	120.19 (19)	N1—C14—C15	119.7 (2)
C6—C5—C4	119.57 (19)	N1—C14—H14	120.2
C6—C5—C10	116.79 (19)	C15—C14—H14	120.2
C4—C5—C10	123.60 (19)	C14—C15—C11	118.5 (2)
C5—C6—C1	121.1 (2)	C14—C15—H15	120.7
C5—C6—H6	119.5	C11—C15—H15	120.7
C1—C6—H6	119.5	O9—C16—O10	125.4 (2)
O1—C7—O2	126.2 (2)	O9—C16—C11	121.5 (2)
O1—C7—C1	116.06 (19)	O10—C16—C11	113.1 (2)
			
C6—C1—C2—C3	0.1 (3)	C3—C2—C8—O4	23.1 (3)
C7—C1—C2—C3	−177.2 (2)	C1—C2—C8—O4	−157.40 (19)
C6—C1—C2—C8	−179.41 (19)	C3—C4—C9—O5	−174.1 (2)
C7—C1—C2—C8	3.3 (3)	C5—C4—C9—O5	4.3 (3)
C1—C2—C3—C4	−0.6 (3)	C3—C4—C9—O6	4.6 (3)
C8—C2—C3—C4	178.9 (2)	C5—C4—C9—O6	−177.0 (2)
C2—C3—C4—C5	0.3 (3)	C6—C5—C10—O7	77.3 (3)
C2—C3—C4—C9	178.74 (19)	C4—C5—C10—O7	−100.5 (3)
C3—C4—C5—C6	0.4 (3)	C6—C5—C10—O8	−97.4 (2)
C9—C4—C5—C6	−178.04 (19)	C4—C5—C10—O8	84.8 (3)
C3—C4—C5—C10	178.15 (19)	C15—C11—C12—C13	0.8 (4)
C9—C4—C5—C10	−0.3 (3)	C16—C11—C12—C13	−178.9 (2)
C4—C5—C6—C1	−0.9 (3)	C14—N1—C13—C12	−0.7 (4)
C10—C5—C6—C1	−178.77 (19)	C11—C12—C13—N1	0.1 (4)
C2—C1—C6—C5	0.6 (3)	C13—N1—C14—C15	0.3 (4)
C7—C1—C6—C5	178.1 (2)	N1—C14—C15—C11	0.6 (4)
C6—C1—C7—O1	64.6 (3)	C12—C11—C15—C14	−1.2 (4)
C2—C1—C7—O1	−118.1 (2)	C16—C11—C15—C14	178.6 (2)
C6—C1—C7—O2	−112.4 (2)	C15—C11—C16—O9	−171.1 (3)
C2—C1—C7—O2	64.9 (3)	C12—C11—C16—O9	8.7 (4)
C3—C2—C8—O3	−157.6 (2)	C15—C11—C16—O10	9.1 (3)
C1—C2—C8—O3	21.9 (3)	C12—C11—C16—O10	−171.2 (2)

### Hydrogen-bond geometry (Å, º)

**Table:**

*D*—H···*A*	*D*—H	H···*A*	*D*···*A*	*D*—H···*A*
O4—H4*O*···O3^i^	0.84 (3)	1.82 (3)	2.654 (3)	176 (3)
O6—H6*O*···O1^ii^	0.85 (5)	1.69 (5)	2.534 (3)	174 (5)
O8—H8*O*···O1*W*	0.85 (5)	1.79 (5)	2.634 (3)	171 (6)
O10—H10*O*···O2^iii^	0.85 (4)	1.78 (4)	2.625 (3)	172 (4)
N1—H1*N*···O5^iv^	0.89 (4)	1.86 (4)	2.711 (3)	160 (4)
O1*W*—H1*W*···O2^v^	0.85 (2)	2.16 (2)	2.957 (3)	156 (3)
O1*W*—H2*W*···O2^iii^	0.85 (1)	2.05 (2)	2.853 (3)	158 (4)
C6—H6···O7^v^	0.95	2.40	3.267 (3)	151
C12—H12···O9^vi^	0.95	2.35	3.179 (4)	146
C14—H14···O6^vii^	0.95	2.40	3.300 (4)	159

Symmetry codes: (i) −*x*+1, −*y*+1, −*z*+2; (ii) *x*+1, *y*, *z*; (iii) *x*, *y*, *z*−1; (iv) *x*−1, *y*, *z*; (v) −*x*+1, −*y*+2, −*z*+1; (vi) −*x*, −*y*+2, −*z*; (vii) −*x*+1, −*y*+1, −*z*+1.

## References

[bb1] Almarsson, Ö. & Zaworotko, M. J. (2004). *Chem. Commun.* pp. 1889–1896. 10.1039/b402150a 15340589

[bb2] Arman, H. D. & Tiekink, E. R. T. (2013*a*). *J. Chem. Crystallogr.* **43**, 134–137.

[bb3] Arman, H. D. & Tiekink, E. R. T. (2013*b*). *Z. Kristallogr. Cryst. Mat* **228**, 10.1524/zkri.2013.1612.

[bb4] Brandenburg, K. (2006). *DIAMOND* Crystal Impact GbR, Bonn, Germany.

[bb5] Chemaxon (2009). *MarvinSketch.* www.chemaxon.com.

[bb6] Dos Santos, L. H. R., Rodrigues, B. L., Idemori, Y. M. & Fernandes, N. G. (2012). *J. Mol. Struct* **101**4, 102–109.

[bb7] Farrugia, L. J. (2012). *J. Appl. Cryst.* **45**, 849–854.

[bb8] Higashi, T. (1995). *ABSCOR* Rigaku Corporation, Tokyo, Japan.

[bb9] Molecular Structure Corporation & Rigaku (2005). *CrystalClear* MSC, The Woodlands, Texas, USA, and Rigaku Corporation, Tokyo, Japan.

[bb10] Sheldrick, G. M. (2008). *Acta Cryst.* A**64**, 112–122. 10.1107/S0108767307043930 18156677

[bb11] Takusagawa, F. & Shimada, A. (1976). *Acta Cryst.* B**32**, 1925–1927.

[bb12] Westrip, S. P. (2010). *J. Appl. Cryst.* **43**, 920–925.

